# Long-Term Trends in Visibility and at Chengdu, China

**DOI:** 10.1371/journal.pone.0068894

**Published:** 2013-07-18

**Authors:** Qiyuan Wang, Junji Cao, Jun Tao, Nan Li, Xiaoli Su, L. W. Antony Chen, Ping Wang, Zhenxing Shen, Suixin Liu, Wenting Dai

**Affiliations:** 1 Department of Environmental Science and Engineering, Xi’an Jiaotong University, Xi’an, China; 2 Key Lab of Aerosol Science & Technology, SKLLQG, Institute of Earth Environment, Chinese Academy of Sciences, Xi’an, China; 3 Institute of Global Environmental Change, Xi’an Jiaotong University, Xi’an, China; 4 South China Institute of Environmental Sciences, SEPA, Guangzhou, China; 5 Division of Atmospheric Sciences, Desert Research Institute, Reno, Nevada, United States of America; University of Milano-Bicocca, Italy

## Abstract

Long-term (1973 to 2010) trends in visibility at Chengdu, China were investigated using meteorological data from the U.S. National Climatic Data Center. The visual range exhibited a declining trend before 1982, a slight increase between 1983 and 1995, a sharp decrease between 1996 and 2005, and some improvements after 2006. The trends in visibility were generally consistent with the economic development and implementation of pollution controls in China. Intensive PM_2.5_ measurements were conducted from 2009 to 2010 to determine the causes of visibility degradation. An analysis based on a modification of the IMPROVE approach indicated that PM_2.5_ ammonium bisulfate contributed 27.7% to the light extinction coefficient (*b_ext_*); this was followed by organic mass (21.7%), moisture (20.6%), and ammonium nitrate (16.3%). Contributions from elemental carbon (9.4%) and soil dust (4.3%) were relatively minor. Anthropogenic aerosol components (sulfate, nitrate, and elemental carbon) and moisture at the surface also were important determinants of the aerosol optical depth (AOD) at 550 nm, and the spatial distributions of both *b_ext_* and AOD were strongly affected by regional topography. A Positive Matrix Factorization receptor model suggested that coal combustion was the largest contributor to PM_2.5_ mass (42.3%) and the dry-air light-scattering coefficient (47.7%); this was followed by vehicular emissions (23.4% and 20.5%, respectively), industrial emissions (14.9% and 18.8%), biomass burning (12.8% and 11.9%), and fugitive dust (6.6% and 1.1%). Our observations provide a scientific basis for improving visibility in this area.

## Introduction

Visibility, a primary index of urban air quality [1], has been deteriorating in China over the past 50 or more years [2]. Poor visibility is linked to human disease [3], and it also significantly impacts tourism and landscape preservation [4]. Visibility impairment is caused by the scattering and absorption of light by particles and gases, and it is a complex issue because many factors can affect it, often non-linearly. These include concentrations, sizes, and composition of particulate matter (PM) as well as meteorological conditions [1]. Sulfate and elemental carbon in PM with aerodynamic diameters ≤2.5 µm (PM_2.5_) are usually the main chemical species contributing to visibility degradation in urban areas [5], [6]. Meteorological factors such as relative humidity and wind speed can influence the concentrations and optical properties of PM_2.5_ as well, thereby contributing to the visibility degradation [7], [8]. Numerous visibility studies, involving a variety of topics from haze formation mechanisms to long-term trends, have been conducted for the rapidly developing Beijing, Pearl River Delta (PRD), and Yangtze River Delta (YRD) regions of China (e.g., [9], [10], [11]). Visibility studies in Southwestern China, including the megacity of Chengdu, on the other hand, are rather limited.

Chengdu, the capital of Sichuan Province, is located in the western portion of the Sichuan Basin (see Fig. S1), and it is considered to be one of the four regions in China most seriously affected by haze. The basin, surrounded by mountains and a plateau that is over 4 km in height, is sheltered from westerly winds and subject to thermal inversions and stagnation; these factors limit the dispersion of locally generated pollutants [12]. Chengdu has a population of ∼11 million and an area of ∼1.2×10^4^ km^2^, and its gross domestic product (GDP) accounts for ∼31% of the GDP for the province. With rapid economic growth and increasing anthropogenic emissions, PM pollution has become one of the primary environmental concerns for the region [13].

In the present study, datasets for visual range (VR, plural VRs) were used to investigate visibility trends in Chengdu from 1973 to 2010. Regional-scale air pollution was studied with light extinction (*b_ext_*, plural *b_ext_*’s) and aerosol optical depth (AOD, AODs) measurements across the Sichuan Basin. Furthermore, the causes of visibility impairment were evaluated through an intensive PM_2.5_ speciation monitoring study and receptor modeling. As many symbols and acronyms are used in this paper, a summary of them is provided in in Table 1.

**Table 1:** Summary of the abbreviations and acronyms used in this study.

Abbreviation or Acronym^a^	Definition
PM	Particulate matter
VR	The farthest distance at which human eye can distinguish a target against a background
*b_ext_*	Attenuation of the incident light by scattering and absorption as it traverses
AOD	Integrated *b_ext_* over a vertical column of unit cross section from the surface to the top of the atmospheres
MODIS	Moderate Resolution Imaging Spectroradiometer
OC	Organic carbon
EC	Elemental carbon
OM	Organic matter
Optical *b_sp,dry_*	Dry particle light scattering coefficient measured by nephelometer at 520 nm
Optical *b_ext_*	Estimated from Koschmieder equation of 3.912/VR
PMF	Positive matrix factorization model
Chemical *b_ext_*	Estimated from chemical species such as sulfate, nitrate, OM, EC, and soil dust based on IMPROVE equation
Chemical *b_sp,dry_*	Estimated from chemical species such as sulfate, nitrate, OM, and soil dust based on IMPROVE equation at dry condition

a In most cases, plurals are formed by adding the letter “s” to the singular form. The exceptions are abbreviations with subscripts.

## Data and Analytical Methods

### 2.1. Meteorological and Aerosol Data

Daily VR observations for a station in Chengdu covering the period from 1973 to 2010 have been archived by the U.S. National Climatic Data Center (NCDC), and these observations constitute the main database for this study. The daily VRs were obtained by averaging a minimum of four synoptic observations per day. Observations with missing codes and those showing precipitation and high (>90%) RH were excluded from the long-term trend analysis. Additionally, daily VRs from 24 synoptic stations in Sichuan Province and Chongqing (see Fig. S1) for March 2009 to February 2010 were retrieved for assessing spatial uniformity. Daily optical *b_ext_*’s were estimated from the well-known Koschmieder equation [14]:
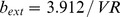
(1)


The Moderate-Resolution Imaging Spectroradiometer (MODIS) is payload instrument that has been deployed on the Terra (in 1999) and Aqua (since 2002) satellites operated by the U.S. National Aeronautics and Space Administration (NASA). Both satellites are in sun-synchronous, near-polar, circular orbits, but differences in their orbits lead to different views for a given location. Aqua crosses the equator at nearly 13∶30 local time, and the AODs, which represent the column-integrated aerosol extinction and reflect surface aerosol concentrations [15], from Aqua were retrieved for this study. MODIS reports AODs in the middle of the visible spectrum (λ = 550 nm) at ∼10 km resolution (at nadir view) with the aerosol algorithm C005-L assuming a dark surface [16].

To characterize the composition of the aerosol, a total of 115 pairs of PM_2.5_ samples were collected on the rooftop (∼20 m above ground level) of the Institute of Plateau Meteorology building in Chengdu (30.67°N, 104.02°E; Fig. S1). Twenty-four-hour PM_2.5_ samples were collected daily from 10∶00 local standard time (LST) to 10∶00 the next day using two battery-powered mini-volume samplers (Airmetrics, Oregon, USA), which operated a flow rate of 5 L min^−1^. One sampler was equipped with 47 mm Teflon® filters (Whatman Limited, Maidstone, UK) for elemental analysis while the other sampler was used with 47 mm quartz-fiber filters (QM/A; Whatman, Middlesex, UK) for water-soluble ions, organic carbon (OC), and elemental carbon (EC) analyses. The samples were grouped into four seasons as follows: spring (31 pairs from April 18 to May 18), summer (32 pairs from July 5 to August 6), and autumn (31 pairs from October 26 to November 26) in 2009 and winter (21 pairs from February 8 to 28) in 2010.

Elemental concentrations, including those of Al, Ca, Mg, Ti, Mn, S, As, Br, Pb, Cu, and Zn, were determined by Energy Dispersive X-Ray Fluorescence (ED-XRF) spectrometry (Epsilon 5 ED-XRF, PANalytical B.V., Netherlands). Details for this procedure have been described in a previous publication [17]. Four inorganic ions (NO_3_^−^, SO_4_^2−^, NH_4_^+^, and K^+^) were analyzed using ion chromatography (IC, Dionex 600, Dionex, Sunnyvale, CA). Anions were analyzed using an ASII-HC column (Dionex) and 20 mM potassium hydroxide as the eluent. Cations were determined using a CS12A column (Dionex) with 20 mM methanesulfonic acid as the eluent. Quartz-fiber filters were preheated to 800°C for 3 h before sampling and the samples were analyzed for carbonaceous species (i.e., OC, EC and carbon fraction) using the IMPROVE A thermal/optical reflectance protocol [18] and a DRI Model 2001 Carbon Analyzer (Atmoslytic Inc., Calabasas, CA, USA). Detailed analytical procedures have been given elsewhere [19], [20].

Five-minute average dry-particle, light-scattering coefficients (*b_sp,dry_*’s, singular *b_sp,dry_*) were continuously determined with the use of an Aurora-1000 single wavelength integrating nephelometer at the wavelength of 520 nm (Ecotech, Melbourne, Australia). A processor-controlled heating system automatically maintained the RH at <60% in the chamber. Span calibration was carried out before sampling period using a haloalkane R-134 reference gas, while zero calibration was performed every two days with particle-free air to account for Rayleigh scattering.

### 2.2. Trend Analysis

A regression model based on the least squares method was used to characterize the long-term trends in VR. The temporal variation in VR was assumed to follow the equation:
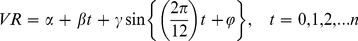
(2)where *t* denotes the elapsed month from a starting time, and *n* is the number of months of interest. The sine term represents a systematic cyclical seasonal variation with phase angle *φ*. The term *β* represents the de-seasonalized monthly rate of change in VR. The annual rate of change is defined by 12*β*.

Ridit analysis can be used to estimate the probability that a visibility observation during a given period is better or worse than a reference visibility distribution [21]. Eq. 3 defines the calculation of ridits with respect to a reference distribution, where *f_A_(v)* represents the probability-density function of visibility observations for a given period *A*, and *f_R_(v′)* is the reference visibility-density distribution. *F_A_* and *F_R_* denote the respective cumulative distribution functions of *f_A_* and *f_R_*, and here *max* is the maximum VR. The probability that an observation from distribution *A* will exceed an observation from distribution *R* is given by:
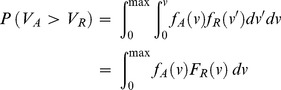
(3)


In this study, ridits were used to compare yearly VR observations with those for the entire 38 year study period. The ridit for each year was estimated by partitioning the VR intervals based on data availability and representing the distributions by histograms. Let *f_Ai_* and *f_Ri_* represent the relative frequencies of the *i*th subinterval for the two distributions, then the mean ridit is calculated by:
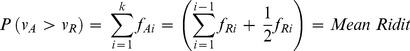
(4)here *f_Ai_* = *n_i_*/*n*, *n_i_* is the number of observations in visibility category *i*, and *n* is the total number of observations, both for distribution *A*. For the reference distribution, *f_Ri_* = *N_i_*/*N* is defined analogously to *f_Ai_*, but *k* sub-divisibions are made. Five VR bins were chosen: 0 to 4.9 km, 5 to 9.9 km, 10 to 14.9 km, 15 to 19.9 km, and >20 km. Ridit scores greater than 0.5 indicate that the VR for a particular year is better than that for the entire period while the opposite is true for ridit scores less than 0.5 [21].

### 2.3. Chemical *b_ext_* Calculation

The revised IMPROVE chemical *b_ext_* equation [22] is
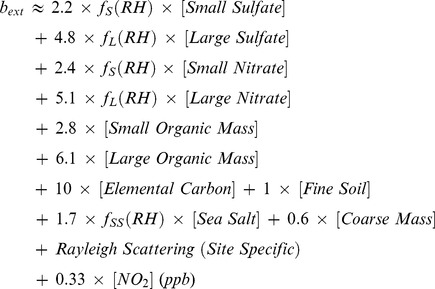
(5)


The apportionment of total concentration of sulfate into the small and large size fractions is accomplished using the following equations [23]:
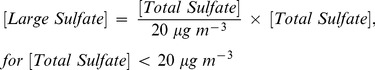
(6)

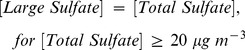
(7)

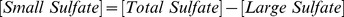
(8)


Similar equations are used to separate total nitrate and organic mass (OM) concentrations into small and large size fractions. As Chengdu is an inland city, the concentration of sea salt is low and for our purposes can be ignored. Moreover, the contributions of coarse mass, NO_2_, and Rayleigh scattering to *b_ext_* have been found to be minor [24], [25], and therefore, they also excluded from the analysis.

### 2.4. Receptor Model Source Apportionment

A Positive Matrix Factorization (PMF) model was used to assess the aerosol sources that contribute to visibility degradation. The principles of PMF have been described in detail elsewhere [26], and the US EPA PMF 3.0 version, which has been widely used in regulatory assessments, was used for our study. The chemical data for the daily PM_2.5_ samples was used for the PMF analysis, and the dataset was composed of the concentrations of 11 elements, water-soluble potassium (K^+^), OC, and EC. The concentrations and signal-to-noise ratios for these analytes are summarized in Table S1.

PMF is a descriptive model, and as such there are no objective criteria for choosing the optimum number of factors that should retained [27]. For our study, solutions with four to seven factors were explored. Each solution converged from random starting points, and a five-factor solution was selected for discussion here because it offered the best interpretability. The frequency distribution of the scaled-fit residuals for each species in the five-factor solution was concentrated between −2 and +2, and this attests to a good model fit. The factor profiles and the daily contributions of the factors were both calculated by our PMF model. Linear regression analysis was then used to estimate the source contributions to PM_2.5_ and optical *b_sp,dry_*.

## Results and Discussion

### 3.1. Long-term Trend in Visibility

**Figure 1:**
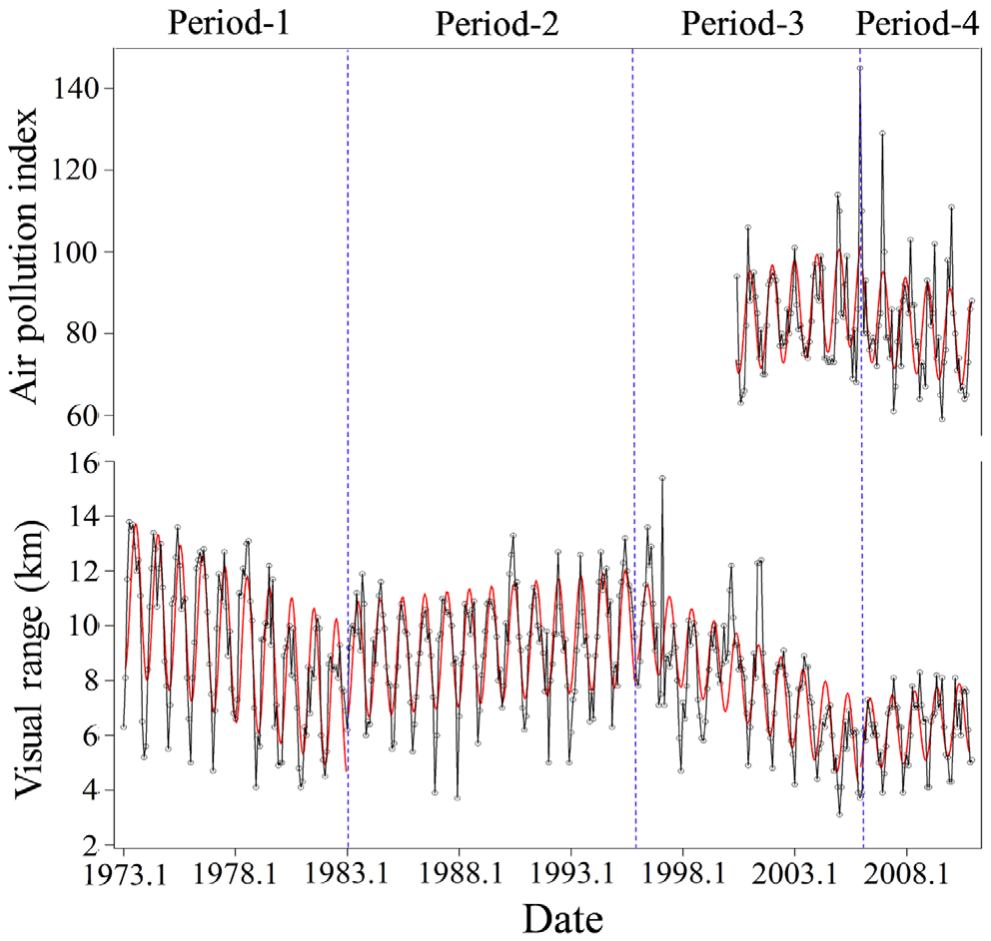
Thirty-eight year trend of monthly average visual range measurements from January 1973 to December 2010 and air pollution index from June 2000 to December 2010 in Chengdu, China. The red solid lines indicate the long-term trends determined by a regression model based on the least squares method.

The 38-year trend in VR from 1973 to 2010 in Chengdu is shown in Fig. 1, and the trend parameters estimated by the regression model (Eq. 2) are summarized in Table 2. The monthly-average VR varied from 3.1 to 15.4 km, with a 38-year average of 8.5±3.9 km; this is at the lower end of the range reported for several large Chinese cities (8.2 at Shenyang to 23.3 Changzhou at km) [11], [28], [29] as shown in the Table S2.

**Table 2:** Coefficients of the regression model for visual range (VR) and air pollution index (API) during Period-1 (1973–1982), Period-2 (1983–1995), Period-3 (1996–2005), and Period-4 (2006–2010) in Chengdu.

Time	Average	S.D.^a^	n^b^	α	β	γ	φ	r^c^	R^d^
Visual Range (km)									
Period-1	9.18	2.65	120	11.11	−0.032	2.81	−1.35	0.86	−0.38
Period-2	9.27	1.92	156	7.67	0.008	2.17	−1.18	0.82	0.01
Period-3	7.89	2.22	120	20.41	−0.037	−1.58	−4.24	0.77	−0.44
Period-4	6.24	1.20	60	1.39	0.011	1.32	−1.10	0.78	0.13
Air Pollution Index									
Period-3^e^	84.78	13.09	67	46.17	0.097	−12.47	4.45	0.66	1.16
Period-4	81.37	13.12	60	130.25	−0.14	−11.64	4.62	0.67	−1.68

a Standard deviation.

b Sample number of the monthly average values of each VR and API.

c Correlation coefficient.

d Annual rate of change: R = 12*β* (km yr^−1^ for VR for API yr^−1^).

e Period-3 for the API was from 2000–2005.

The overall trend of VR during the whole 38 years showed a declining rate of −0.08 km yr^−1^. Based on the patterns shown in Fig. 1, the VR records were separated into four periods as follows: 1973 to 1982 (Period-1), 1983 to 1995 (Period-2), 1996 to 2005 (Period-3), and 2006 to 2010 (Period-4). Period-1 showed a clear decreasing trend in VR, from 10.8 km in 1973 to 7.7 km in 1982; this is equivalent to a rate of −0.38 km yr^-1^ (12*β*). VR degradation during this period may be associated with the national economic recovery after a long period of stagnation during the 1960s. Similar decreases in VR were found in most regions of the Sichuan Basin and southeastern China during this period [30], [31].

As a consequence of the strong industrial growth in the 1990s, acid precipitation became a problem in China [32], and this forced the government to enact stringent series of pollution controls. These included limits on the sulfur contents of fuels and reductions in the SO_2_ emissions from large power plants. VR showed a slight improvement during Period-2 (+0.01 km yr^−1^) more than likely due to these pollution controls, at least in part. However, a rapid decrease in VR was again observed during Period-3, from 10.4 km in 1996 to 5.3 km in 2005, a rate of −0.44 km yr^−1^.

The decrease in VR during Period-3 was inversely related to the China Air Pollution Index (API, available online at http://datacenter.mep.gov.cn), which increased during this period and is calculated from the concentrations of PM_10_, SO_2_, and NO_2_ (Fig. 1). Indeed, the VR during Period-3 was anti-correlated with the API−the correlation coefficient for a linear regression between the two variables was −0.43. This shows that visibility decreased as pollution levels increased, and the degradation in visibility during this time is doubtlessly connected to the rapid economic growth and industrial development in China. For instance, the coal consumption in Sichuan increased from 4.9×10^7^ tons in 2000 to 8.5×10^7^ tons in 2005 [33], and the number of civilian motor vehicles increased from 0.76×10^6^ to 1.4×10^6^ [34]. Therefore, the degradation in VR during Period-3 can be explained in large measure by the increased emissions from coal combustion and motor vehicles.

**Figure 2:**
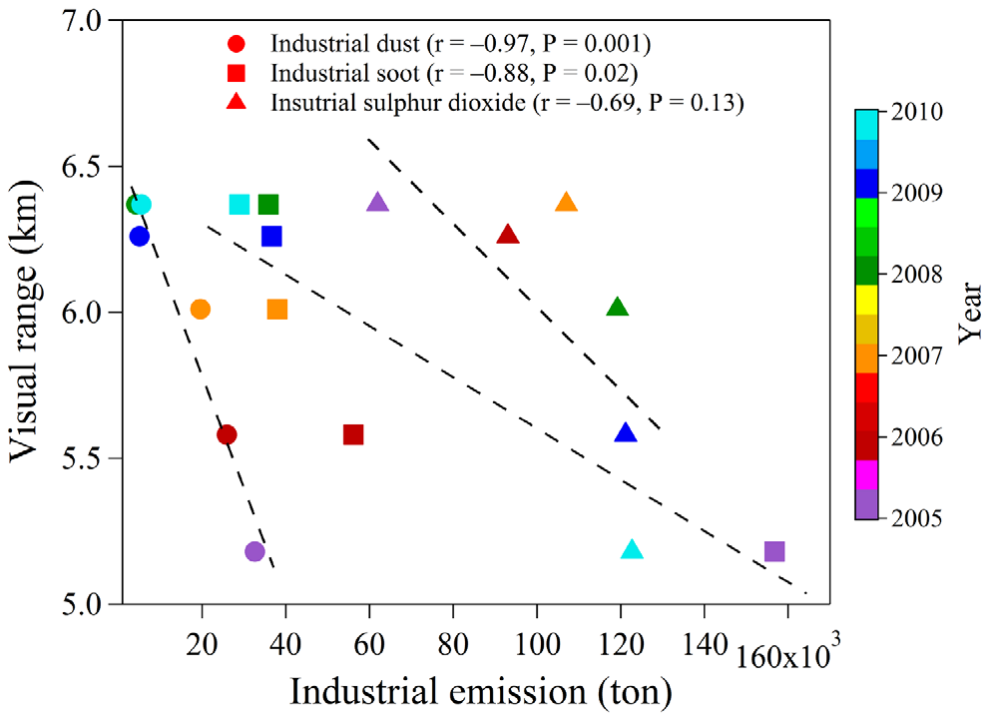
Scatter plot of visual range versus industrial emissions including industrial dust, soot, and sulfur dioxide during 2005–2010. The dash lines represent the linear trend determined by regression. The color bar shows the year for the industrial emissions.

During Period-4, the most recent interval, VR improved slightly from 5.8 km in 2006 to 6.4 km in 2010, equivalent to a rate of increase of 0.13 km yr^−1^. Chengdu is a critical area for the control for acid rain in China’s “Two Control Zones,” and more stringent regulations on coal combustion and SO_2_ emissions have been enacted since the Eleventh Five-Year Plan was implemented in 2006 (available online at http://www.schj.gov.cn). Investments in environment management increased significantly during this period, and the API exhibited a decreasing trend. Fig. 2 shows that VR strongly anti-correlates with industrial dust and soot emissions (r = −0.97 and r = −0.88, respectively), and moderately anti-correlates with industrial SO_2_ emissions (r = −0.69). In fact, the industrial dust, soot and SO_2_ emissions in Chengdu dramatically decreased by factors of 2 to 6 from 2005 to 2010 [35]. Benefits from the national pollution control and regulatory policies are likely reflected in the increasing trend in VR during Period-4; indeed, several other areas in China showed similar improvements in VR during this time [30], [31].

**Figure 3:**
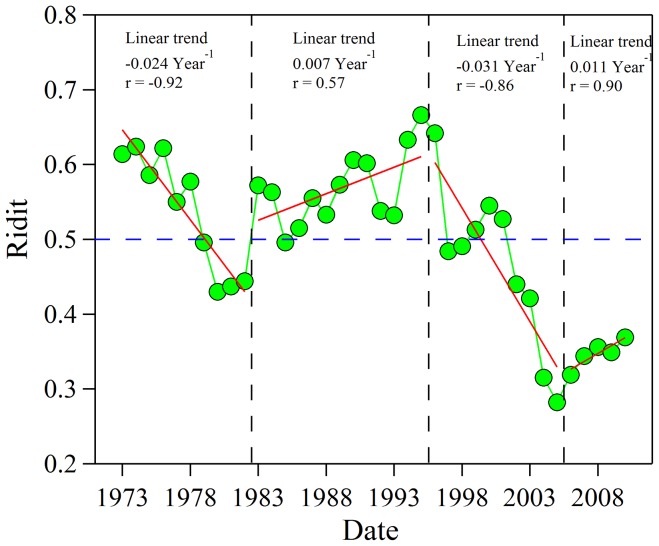
Annual variations of ridit values during 1973–2010 in Chengdu. Ridit values >0.5 mean that the visual range for the year was better than the reference distribution established from the 1973–2010 data; the opposite is true for value <0.5. Solid lines are linear fits of the ridit trends.

The annual ridit values declined at a rate of −0.024 yr^−1^ for Period-1 and −0.031 yr^−1^ for Period-3, but they increased at a rate of 0.007 yr^−1^ and 0.011 yr^−1^ for Periods 2 and 4, respectively (Fig. 3). The annual ridit values before 1978 were greater than 0.5 suggesting that VRs during that time were higher compared with the entire timeframe for the study. The annual ridits were less than 0.5 between 1979 and 1982; but from 1983 to 1995, the ridit values exceeded 0.5, and this consistent with improvements in visibility. After 1997, the annual ridit values were all less than 0.5, except for 1991 to 2001. Although an increasing trend in VR was obvious after the introduction of the Eleventh Five-Year Plan, the average VR was still low compared to historical levels. Selected periods are considered in the following sections to investigate the impacts of specific chemical species and sources on visibility impairment.

### 3.2. Case Study

#### 3.2.1. Spatial distribution of optical *b_ext_* and AOD

**Figure 4:**
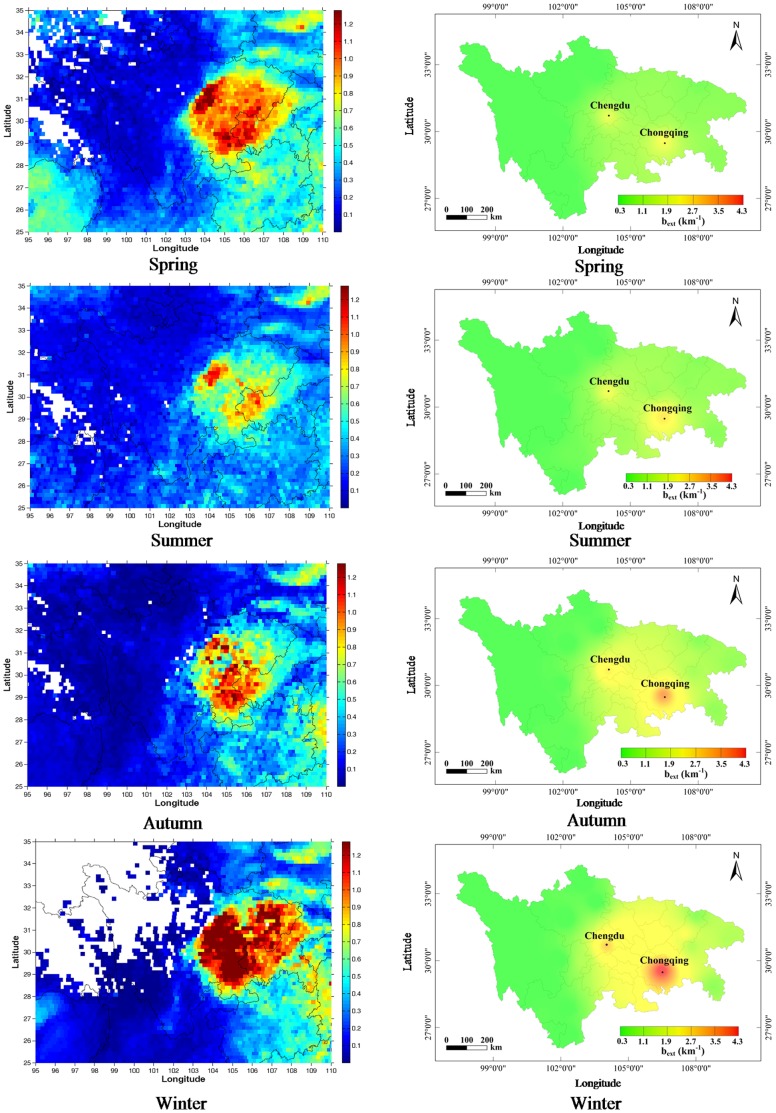
Left panel: Spatial and seasonal distributions of average MODIS/Aqua AOD at 550 nm. Right panel: light extinction coefficient (*b_ext_*) estimated from Koschmieder’s formula over the Sichuan Basin during March 2009 to February 2010.

The spatial distributions of surface-level optical *b_ext_* and columnar AOD over Sichuan Province and the city of Chongqing during four seasons from 2009 to 2010 are presented in Fig. 4. The most distinctive feature in the plots is the apparent influence of terrain on the distributions of *b_ext_* and AOD. In low-lying areas, such as the east-central part of Sichuan Province and Chongqing, both *b_ext_* and AOD generally exhibited high values. In contrast, low values for these variables mostly appeared in mountainous regions west of Sichuan where the elevation is almost 10 times higher than that in the eastern-plain areas. This regional terrain impeded the transport and diffusion of air pollutant into the plains, and it contributed to the heterogeneous patterns in *b_ext_* and AOD. In addition to topography, the large quantities of air pollutants emitted in the eastern part of the study area, which is densely populated and heavily developed, also contributed to the high values of *b_ext_* and AOD in those areas.

As evident in Fig. 4, both *b_ext_* and AOD also displayed significant seasonal variability. Generally, the largest seasonal difference between *b_ext_* and AOD occurred in spring when high AOD values were distributed throughout the basin, but in contrast, high *b_ext_* values were only concentrated around Chengdu and Chongqing. This difference was probably caused by dust events which frequently occur during this time of year [13], [36]. It is worth noting that AOD measures the vertical column-integrated extinction coefficient from the surface to the top of the atmosphere whereas *b_ext_* as used here was calculated from the surface horizontal visibility using Koschmieder’s formula. Consequently, the columnar AOD includes the effects of the dust particles which are mostly transported above 1 km [36] while the surface-level *b_ext_* is less sensitive to that fraction of the aerosol. Except for the spring, both *b_ext_* and AOD exhibited roughly similar seasonal trends, following the sequence of summer<autumn<winter.

In summer, both *b_ext_* and AOD were lower than in autumn or winter. This can be explained to some extent by wet scavenging because precipitation is most frequent summer, accounting for ∼55% of yearly total [35]. Wet deposition is thought to be the main way in which particles are removed from the atmosphere in the area [37], and this shortens the aerosol particles’ atmospheric lifetimes. In autumn, both *b_ext_* and AOD started to increase especially in the southern part of the basin; this was most likely caused by the burning of straw, a practice used to clear agricultural fields. In winter, both *b_ext_* and AOD were at their maximum, and both showed high values throughout the whole basin. These high aerosol loadings were likely the result of higher pollution emissions caused by an increase in energy consumption, especially the burning of coal and biomass for residential heating. Additionally, the occurrence of inversion layers in winter limited the advection of air pollutants, and thus meteorological conditions also probably contributed to the high *b_ext_* and AODs in winter.

#### 3.2.2. Influences of chemical components on *b_ext_* and AOD

As discussed below, secondary inorganic ions (NO_3_^−^, SO_4_^2−^, and NH_4_^+^) were the major light scattering components, and therefore these species were a primary concern for our study. The concentration of NH_4_^+^ was strongly correlated with SO_4_^2−^ and NO_3_^−^, with correlation coefficients of 0.91 and 0.95 (not shown), respectively, suggesting that these three ions were in the form of ammonium sulfate ((NH_4_)_2_SO_4_), ammonium bisulfate (NH_4_HSO_4_) and ammonium nitrate (NH_4_NO_3_). If one assumes that the dominant compounds were NH_4_HSO_4_ and NH_4_NO_3_, the NH_4_^+^ concentrations can be calculated using Eq. 9; alternatively if NH_4_^+^ were in the form of (NH_4_)_2_SO_4_ and NH_4_NO_3_, Eq. 10 would apply [38]:
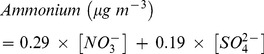
(9)

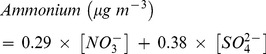
(10)where [NO_3_^−^] and [SO_4_^2−^] represent the mass concentrations of NO_3_^−^ and SO_4_^2−^, respectively.

**Figure 5:**
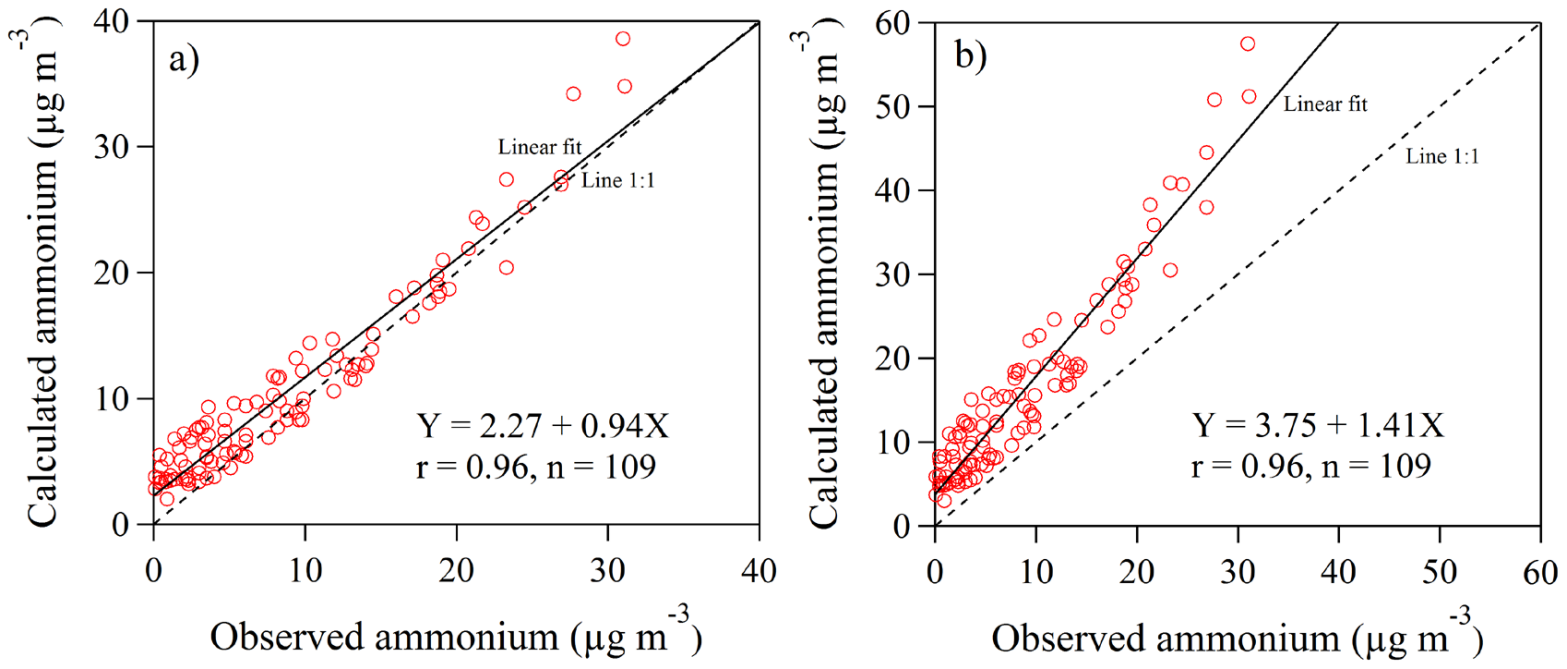
Scatter plots of ammonium calculated from (a) 0.29×[NO_3_^−^] +0.19×[SO_4_^2^^−^] and (b) 0.29×[NO_3_^−^] +0.38×[SO_4_^2^^−^] versus ammonium measured by ion chromatography.

A comparison of the calculated versus the observed NH_4_^+^ concentrations is presented in Fig. 5. The NH_4_^+^ concentrations calculated from both Eq. 9 and 10 showed strong correlations with the observed NH_4_^+^, but the slope from Eq. 9 was closer to unity (0.94, Fig. 5a) than that from Eq. 10 (1.41, Fig. 5b); and this indicates that the three major ions predominantly existed as NH_4_HSO_4_ and NH_4_NO_3_.

The IMPROVE approach (Eq. 5 to 8) was then used to partition the chemical *b_ext_* and *b_sp,dry_* among the measured PM_2.5_ chemical components. The calculation of chemical *b_sp,dry_* was estimated from the loadings of sulfate, nitrate, OM, and soil dust when particles were not influenced by RH, that is, RH <60%. As discussed above, we can assume that NO_3_^−^, SO_4_^2−^, and NH_4_^+^ mainly existed as NH_4_HSO_4_ and NH_4_NO_3_, and therefore, the concentrations of NH_4_HSO_4_ and NH_4_NO_3_ can be calculated by multiplying the SO_4_^2−^ and NO_3_^−^ concentrations by factors of 1.20 and 1.29, respectively. The OM and soil dust fractions were estimated from 1.8×[OC] [39] and [Fe]/0.035 [40], respectively.

The reconstructed chemical *b_ext_* correlated strongly with the measured values (Fig. S2); the slope for a least-squares linear regression was 1.01, with r = 0.88. The reconstructed chemical *b_sp,dry_* correlated even better with the measured *b_sp,dry_* although the slope for that regression was farther from unity (slope = 0.81 and r = 0.96). These results show that the IMPROVE algorithm can provide reasonable estimates for chemical *b_ext_* at Chengdu under both dry and ambient conditions.

**Figure 6:**
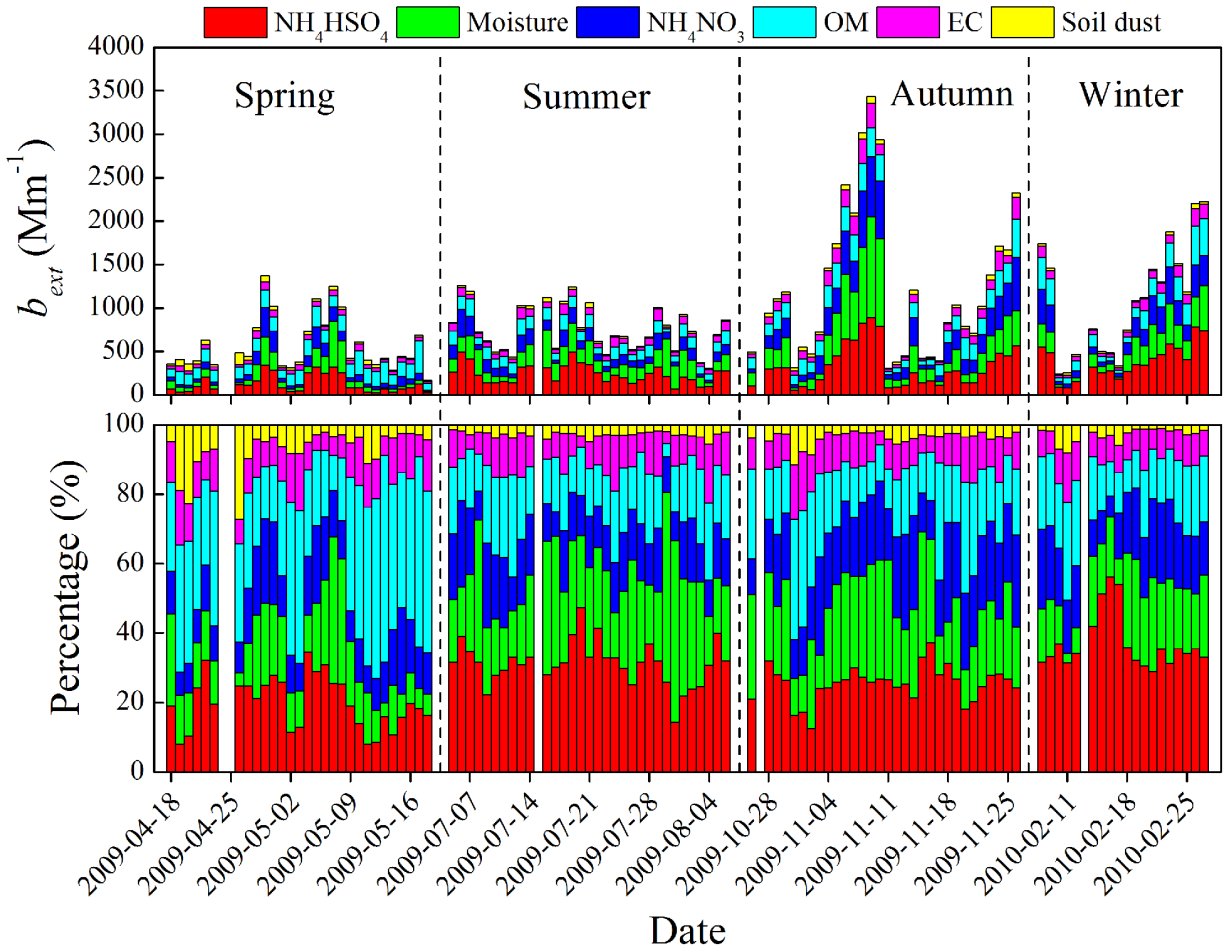
Daily variations of the contributions of PM_2.5_ chemical components and aerosol moisture to the light extinction coefficient (*b_ext_*) for the intensive sampling period based on the revised IMPROVE equation. The aerosol moisture contributions were calculated from *b_ext_* under ambient condition subtracts *b_ext_* under dry condition.

The daily contributions of PM_2.5_ chemical components and aerosol moisture to chemical *b_ext_* that were calculated using the IMPROVE approach are presented Fig. 6. The aerosol moisture contributions, in the form of scattering enhancement factors, were calculated from *b_ext_* under ambient condition subtracts *b_ext_* under dry condition. The average chemical *b_ext_* was the highest in autumn (1224 Mm^−1^), followed by winter (1101 Mm^−1^), summer (760 Mm^−1^), and spring (576 Mm^−1^), with an annual average of 900±623 Mm^−1^. These values were much higher than those observed at Guangzhou (367 Mm^−1^) [41] or Jinan (292 Mm^−1^) [42], but similar to the value of 912 Mm^−1^ reported for Xi’an [24].

In urban atmospheres, aerosol SO_4_^2−^ is formed through the oxidation of SO_2_ by both heterogeneous and homogeneous processes, and it is removed by both dry and wet deposition [43]. On average, NH_4_HSO_4_ was the largest contributor to scattering: it accounted for 27.7% of chemical *b_ext_*, and the greatest effect was in winter (37.0%), followed by summer (31.2%), autumn (25.4%), and spring (20.0%) (Fig. 6). Although industrial SO_2_ emissions, which are the main source for SO_2_ in urban areas, showed a declining trend in Chengdu during the period of the Eleventh Five-Year Plan (2006 to 2010), the SO_2_ emissions have remained high, 6.2×10^4^ tons in 2010 (see Fig. 2). Studies in Xi’an [24], Jinan [42], and Guangzhou [10] have similarly shown that sulfate was the largest contributor to *b_ext_*.

On average OM (21.7%) and moisture (20.6%) contributed roughly similar amounts to *b_ext_*. The contribution of OM was elevated during spring (33.5%), while increased moisture contributions were found in summer (25.0%) and autumn (22.8%) due in part to the greater effects of the higher RH on the major ions. The RH averaged 80% in summer and 77% in autumn, compared to 70% in spring and 73% in winter. The NH_4_NO_3_ contributions to *b_ext_* were relatively consistent throughout the year, with range for the seasonal averages of 13.6 to 19.7%. On average, particle light absorption from EC contributed just 9.4% to *b_ext_*, and an even smaller percentage of *b_ext_* (2.8 to 7.2%) was explained by the loadings of soil dust.

We compared the PM_2.5_ chemical loadings during good and poor visibility conditions by averaging the data for 2.5% of the days with the best visibility (VR >10.5 km, abbreviated as Best 2.5%) and doing the same for the 2.5% of the days with the worst visibility (VR <1.5 km, Worst 2.5%) (Table 3). The PM_2.5_ loadings differed by a factor of 6.3 between the Best 2.5% (63.6 µg m^−3^) and Worst 2.5% days (400.8 µg m^−3^). The concentrations of secondary inorganic ions for the Worst 2.5% were 5.7 to 10.8 times higher than during the Best 2.5%. The concentrations of OM and EC differed by 380% and 644%, respectively, between the two categories. This analysis shows that VR impairment was consistent with elevated loadings of PM_2.5_, especially those of the secondary inorganic ions.

**Table 3:** Average chemical component concentrations and meteorological parameters for the best and worst visual ranges (VRs).

Variable^a^	Best 2.5%^b^ (VR >10.5 km)		Worst 2.5%^c^ (VR <1.5 km)	
	Average	S.D.^d^	Average	S.D.
PM_2.5_	63.6	11.9	400.8	37.5
NO_3_^−^	5.9	0.4	63.8	3.6
SO_4_^2−^	15.9	5.2	91.2	7.5
NH_4_^+^	2.8	1.7	29.9	1.9
K^+^	1.0	0.5	8.5	0.4
OM^e^	15.4	7.3	58.6	2.0
EC^f^	3.6	1.0	23.2	9.5
RH	63.0	4.4	83.7	0.6
WS	1.3	0.2	0.5	0.3
MLD	663.1	83.4	250.9	64.6

a Units: PM_2.5_ and chemical species, µg m^−3^; Relative humidity (RH), %; Wind speed (WS), m s^−1^; Mixed layer depth (MLD), m.

b daily average VR values for the 2.5% least impaired days.

c daily average VR values for the 2.5% most impaired days.

d S.D.: Standard deviation.

e OM: Organic mass = 1.8×OC.

f EC: Elemental carbon.

Table 4 shows the chemical *b_ext_* budget for the aerosol components in the Best 2.5% and Worst 2.5% categories. The average contributions of NH_4_HSO_4_, NH_4_NO_3_, OM, and EC on *b_ext_* on the Worst 2.5% days were 835, 667, 318, and 232 Mm^−1^, respectively; these are 3.5 to 16.1 times higher than those under the Best 2.5% conditions. The effect of moisture on *b_ext_* increased from 30 to 1015 Mm^−1^, an increase of 34.2 times, from the Best 2.5% to Worst 2.5% conditions. The combined moisture (32.4%) and NH_4_HSO_4_ (26.7%) contributions accounted for nearly 60% of *b_ext_* for the Worst 2.5% category. The results support our conclusion that increases in water vapor and large NH_4_HSO_4_ loadings were the main factors leading to visibility degradation in Chengdu on the days with the worst visibility.

**Table 4:** Changes in light extinction (*b_ext_*) budgets for PM_2.5_ components and moisture for the Best 2.5% and the Worst 2.5% visual range observations.

	Best 2.5%^a^			Worst 2.5%^b^		
	Average	S.D.	% of *b_ext_*	Average	S.D.	% of *b_ext_*
NH_4_HSO_4_	133.5	50.7	39.5	834.7	52.8	26.7
NH_4_NO_3_	41.4	2.5	12.3	667.2	21.3	21.3
OM	78.8	44.4	23.3	317.5	10.8	10.1
EC	35.6	10.4	10.5	231.8	94.8	7.4
Soil dust	18.9	2.4	5.6	65.4	14.8	2.1
Moisture	29.7	25.7	8.8	1014.7	143.9	32.4

a Group composed of the 2.5% of the days least impaired visual ranges,

b Group of the 2.5% most impaired days.

**Figure 7:**
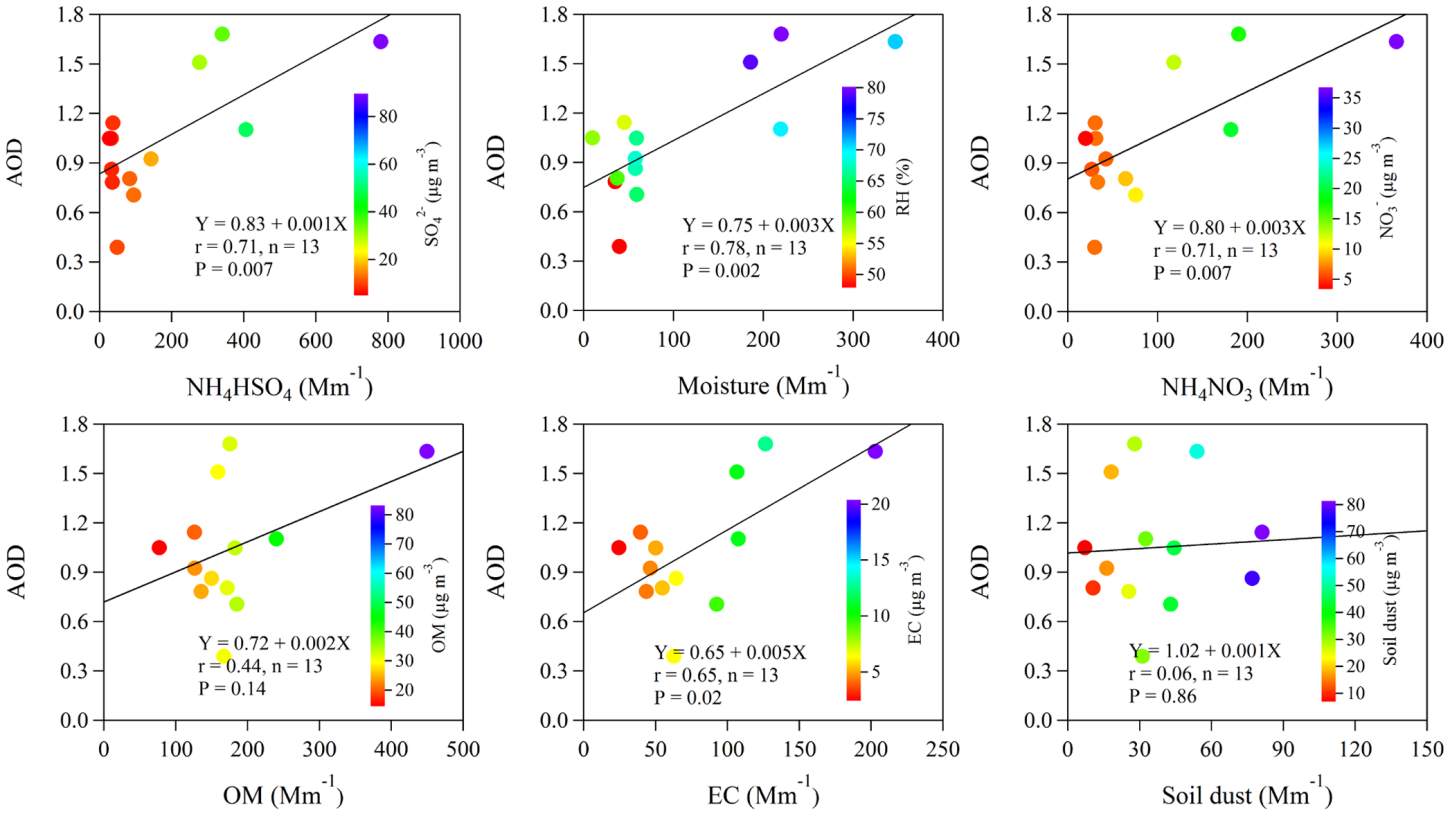
Correlations between AOD at 550 nm and light extinction by PM_2.5_ NH_4_HSO_4_, NH_4_NO_3_, moisture, organic matter (OM), elemental carbon (EC), and soil dust. The color bars indicate the concentrations of the chemical species or relative humidity (RH).

Fig. 7 presents the relationship between AOD at 550 nm and *b_ext_* values calculated for the chemical species and moisture. The *b_ext_*’s for NH_4_HSO_4_, NH_4_NO_3_, and moisture were significantly related to AOD, with probabilities for chance occurrence of less than 5% (p<0.05) and correlation coefficients of 0.71, 0.71, and 0.78, respectively. The correlations between AOD and *b_ext_* caused by OM and EC were weaker, with respective r values of 0.44 and 0.65. The correlation between AOD and *b_ext_* attributable soil dust was not significant, that is, p>0.05. In summary, the AODs were strongly affected by RH, but they also increased as the anthropogenic aerosol loadings increased, especially those of SO_4_^2−^, NO_3_^−^, and EC (Fig. 7).

#### 3.2.3. Source apportionment of PM_2.5_ and VR degradation

**Figure 8:**
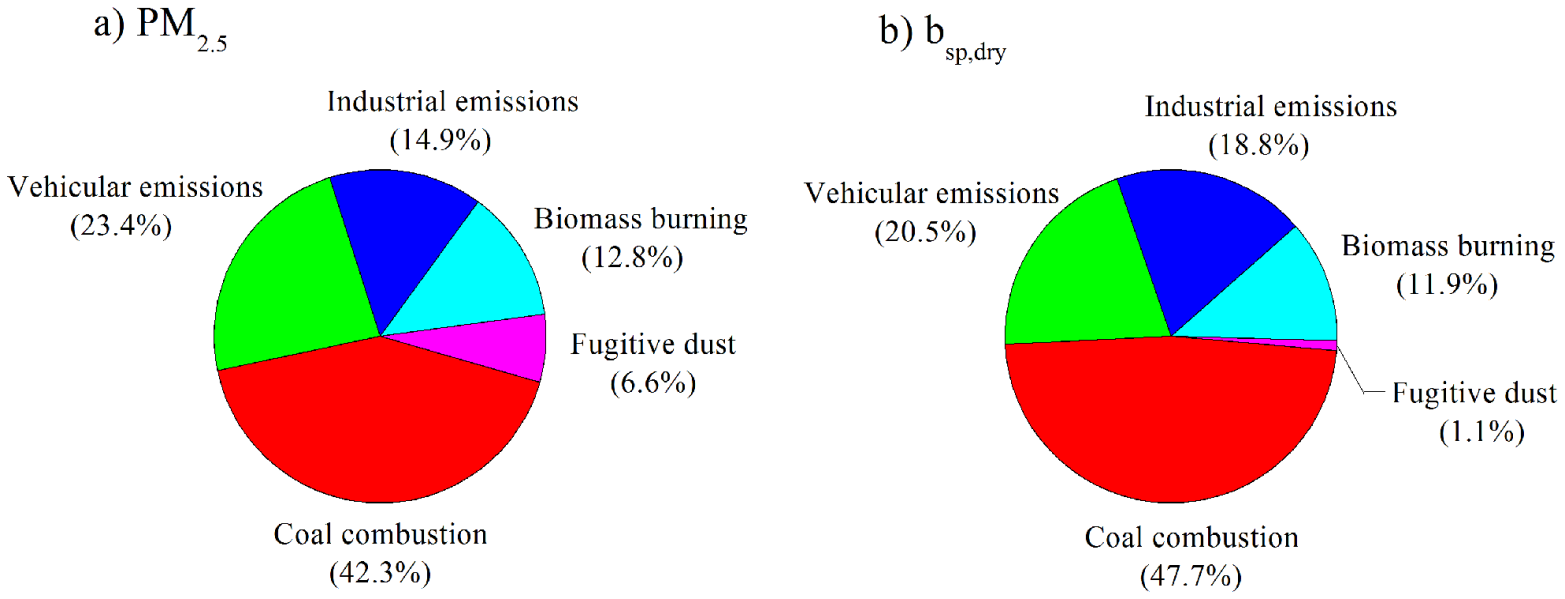
Average source contribution (in percent) for each PMF source factor to PM_2.5_ mass concentration and dry particle light scattering coefficient (*b_sp,dry_*).

The optical *b_ext_*’s estimated from VRs were highly correlated with the optical *b_sp,dry_*’s measured with the use of a nephelometer (Fig. S3). The correlation coefficient for the regression of *b_sp,dry_* on *b_ext_* was 0.88 and the slope was 0.83. Therefore the *b_sp,dry_* can be considered generally representative of the *b_ext_* and of the VR. To further investigate the causes for the visibility degradation, PMF analyses were conducted to apportion the collocated PM_2.5_ chemical and optical *b_sp,dry_* data to source factors; these analyses used data for OC, EC, K^+^, Al, Ca, Mg, Ti, Mn, S, As, Br, Pb, Cu, and Zn. The resulting PMF factor profiles and source attributions are presented in Fig. S4, and Fig. 8 shows the average source contributions.

Factor 1 was enriched in S, Pb, and As, and it was ascribed to coal combustion. This factor accounted for 42.3% of the PM_2.5_ and 47.7% of the *b_sp,dry_*. Energy consumption (including coal, petroleum and gas) in Sichuan Province increased by 38% during the Eleventh Five-Year Plan, from 1.3×10^8^ tons in 2006 to 1.8×10^8^ tons in 2010. Of this, 64.4 to 74.4% was associated coal combustion [33], and it is clear that sizeable improvements in visibility would be realized if greater efforts were made to promote clean energy sources as replacements for coal.

Factor 2 had high loadings of EC, OC, and Br, and this factor was interpreted as motor vehicle emissions. This source accounted for 23.4% and 20.5% of PM_2.5_ and *b_sp,dry_*, respectively. The total number of motor vehicles in Chengdu increased by 41%, from ∼1.7×10^6^ in 2006 to ∼2.4×10^6^ in 2010 [30].

Factor 3 had a relatively high loadings of Zn as well as Mn, As, Br, Pb, and Cu, and this factor was most likely associated with industrial emissions; it accounted for 14.9% of PM_2.5_ and 18.8% of *b_sp,dry_*. Large quantities of industrial dust and soot are emitted during a variety of industrial processes, and the quantities emitted in Chengdu have been estimated to be 5.5×10^3^ and 2.9×10^4^ ton yr^−1^, respectively, for 2010 (see Fig. 2).

Factor 4, was characterized by K^+^ and OC, and this is most consistent with biomass burning emissions. In nearby non-urban areas, wheat straw is burned for cooking year round, and the burning of agricultural fields takes place immediately during the harvest season [44]. This source accounted for 12.8% and 11.9% of PM_2.5_ and *b_sp,dry_*, respectively.

Factor 5 was loaded with Al, Ca, Mg, Ti, and Mn, and it represents fugitive dust. This source accounted for only 6.6% of the PM_2.5_ and even less (1.1%) of the *b_sp,dry_*. This small effect was at least partly due to low wind speeds throughout the year.

### Conclusions

This long-term (1973 to 2010) study of visibility in Chengdu showed that the 38-year average VR was 8.5±3.9 km; it exhibited a declining trend before 1982, increased slightly between 1983 and 1995, decreased sharply between 1996 and 2005, and showed some improvements after 2006. The trends are generally consistent with the development of the national economy and the implementation of pollution.

Analyses of the spatial distributions of optical *b_ext_* and AOD showed high values in the eastern part of Sichuan Province, and this contrasted sharply with the low values retrieved for the western part of the Province. The largest difference in the seasonal variability between the two variables was observed in spring, and this was probably caused dust events because they are common during that time of year. Studies of PM_2.5_ collected during an intensive observing period (April 2009 to February 2010) showed that NH_4_HSO_4_ was the largest contributor to the chemically reconstructed *b_ext_*. Ammonium bisulfate accounted for 27.7% of *b_ext_*, and it was followed by OM (21.7%), moisture (20.6%), and NH_4_NO_3_ (16.3%); EC and soil dust contributed relatively little to extinction (9.4 and 4.3%, respectively). High RHs and large NH_4_HSO_4_ loadings were the main factors leading to visibility degradation under the worst conditions, that is, VR<∼1.5 km, in Chengdu. The results also indicated that AODs at 550 nm were correlated with the concentrations of anthropogenic aerosols (such as sulfate, nitrate, and EC) and with the amount of moisture at the surface.

The PMF receptor model indicated that coal combustion was the dominant contributor to PM_2.5_ and to the *b_sp,dry_* during the intensive observation period (42.3% and 47.7%, respectively); this was followed by vehicular emissions (23.4% and 20.5%), industrial emissions (14.9% and 18.8%), biomass burning (12.8% and 11.9%), and fugitive dust (6.6% and 1.1%).

The results of our analyses and case study will benefit other megacities in China and elsewhere because they provide guidance on how the causes for visibility impairment can be determined. It is also worth reiterating that the visual effects of air pollution are just one consideration. Indeed, the effects of air pollution on public health and more broadly on the environment are of equal if not greater importance than the impairment of visibility. Finally, our studies relating specific sources of PM_2.5_ to reductions in visibility provide information that can be used to address the problem of haze pollution in Chengdu; the results also are likely to be relevant for other Chinese urban areas.

## Supporting Information

Figure S1 Locations of cities and visibility observation stations in Sichuan Province and Chongqing as well as the intensive sampling site in Chengdu. The yellow rectangle represents the Sichuan Basin.
(TIF)

Figure S2 (a and b) Scatter plots of reconstructed chemical light extinction versus observed light extinction coefficient (bext) and dry particle light scattering coefficient (bsp,dry). Reconstructed bext and bsp,dry were calculated using a revised IMPROVE algorithm.
(TIF)

Figure S3 Scatter plots of the dry particle light scattering (bsp,dry) coefficient measured with a nephelometer versus light extinction coefficient (bext) estimated from the Koschmieder equation.
(TIF)

Figure S4 Source profiles for the five sources identified by the Positive Matrix Factorization (PMF) model during the intensive sampling period at Chengdu. Left Y-axis represents the percentage that each source contributes to each species. Right Y-axis represents the relative concentration that each source contributes to the species.
(TIF)

Table S1 Summary of the concentrations and signal-to-noise ratios (S/N) for the analytes used in PMF analysis.
(DOCX)

Table S2 Visual ranges (VR) for selected cities in China.
(DOCX)
