# Supplementary figures and images for: Long-Term Trends in Visibility and at Chengdu, China
Source: PLoS One. 2013 Jul 18;8(7):e68894. doi: 10.1371/journal.pone.0068894 (PMC3715545; doi:10.1371/journal.pone.0068894)

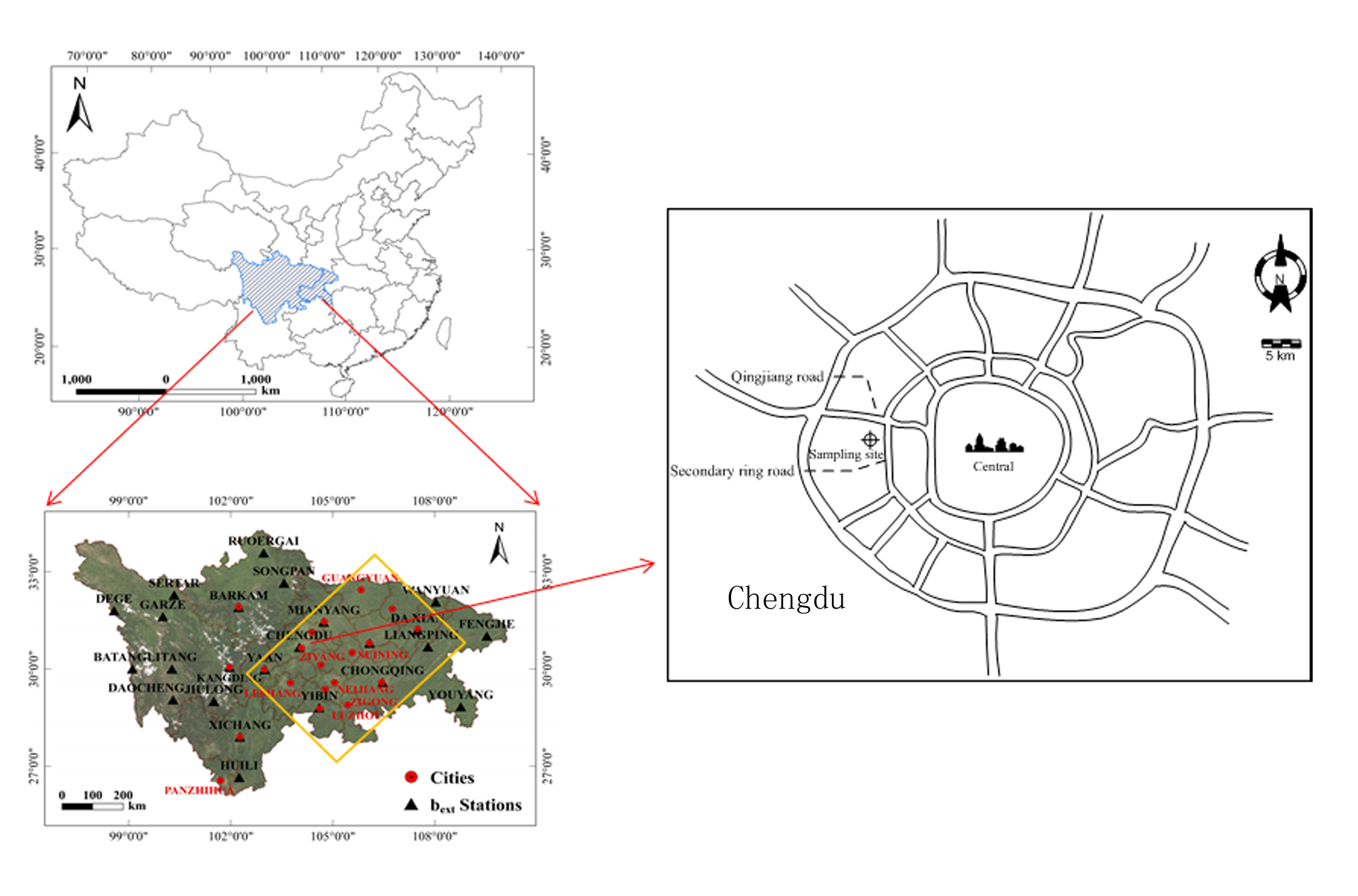

Supplement: Figure S1 — Locations of cities and visibility observation stations in Sichuan Province and Chongqing as well as the intensive sampling site in Chengdu. The yellow rectangle represents the Sichuan Basin. (TIF) [file pone.0068894.s001.tif]

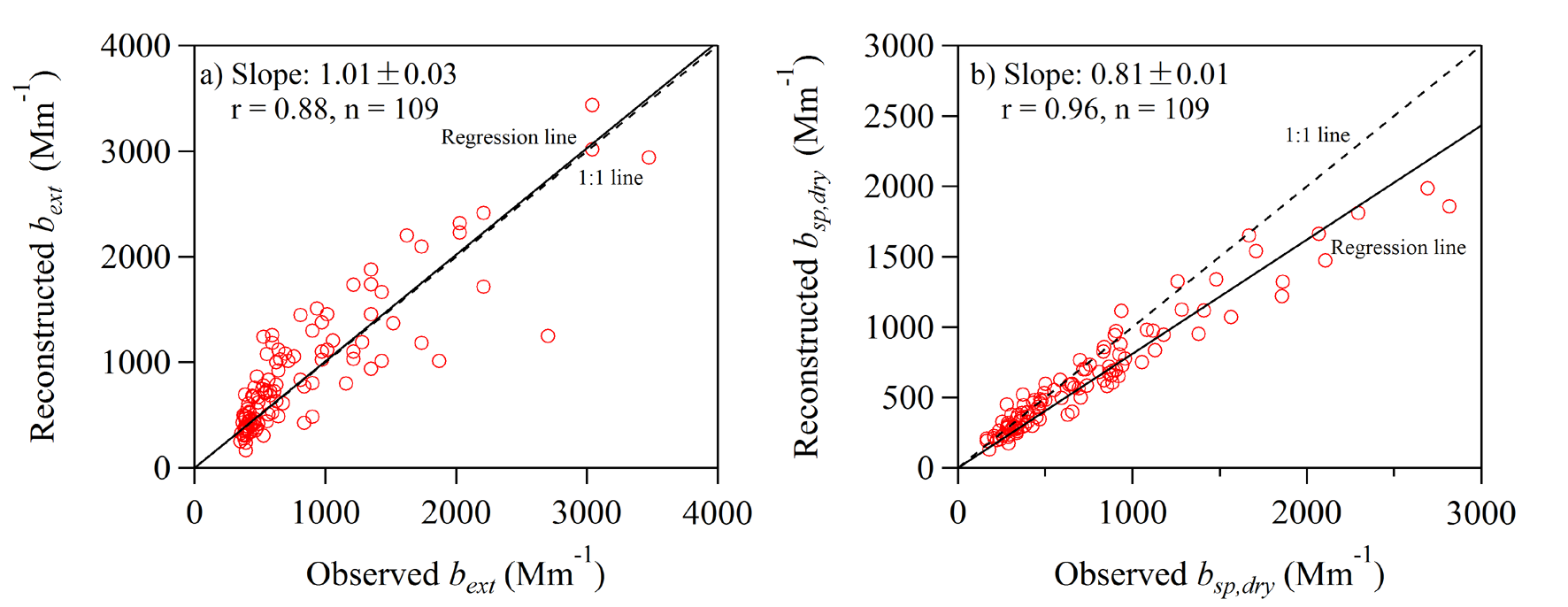

Supplement: Figure S2 — (a and b) Scatter plots of reconstructed chemical light extinction versus observed light extinction coefficient (bext) and dry particle light scattering coefficient (bsp,dry). Reconstructed bext and bsp,dry were calculated using a revised IMPROVE algorithm. (TIF) [file pone.0068894.s002.tif]

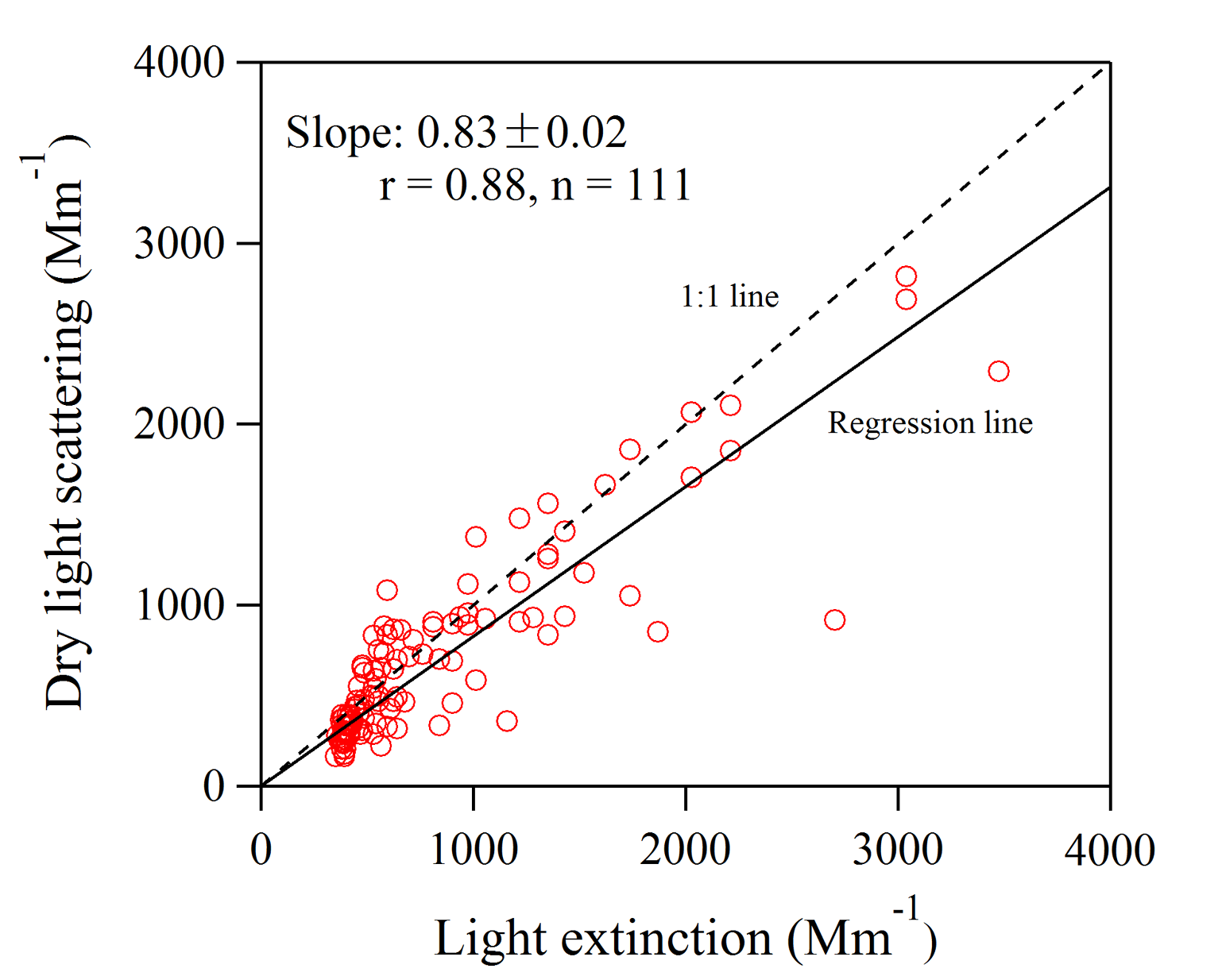

Supplement: Figure S3 — Scatter plots of the dry particle light scattering (bsp,dry) coefficient measured with a nephelometer versus light extinction coefficient (bext) estimated from the Koschmieder equation. (TIF) [file pone.0068894.s003.tif]

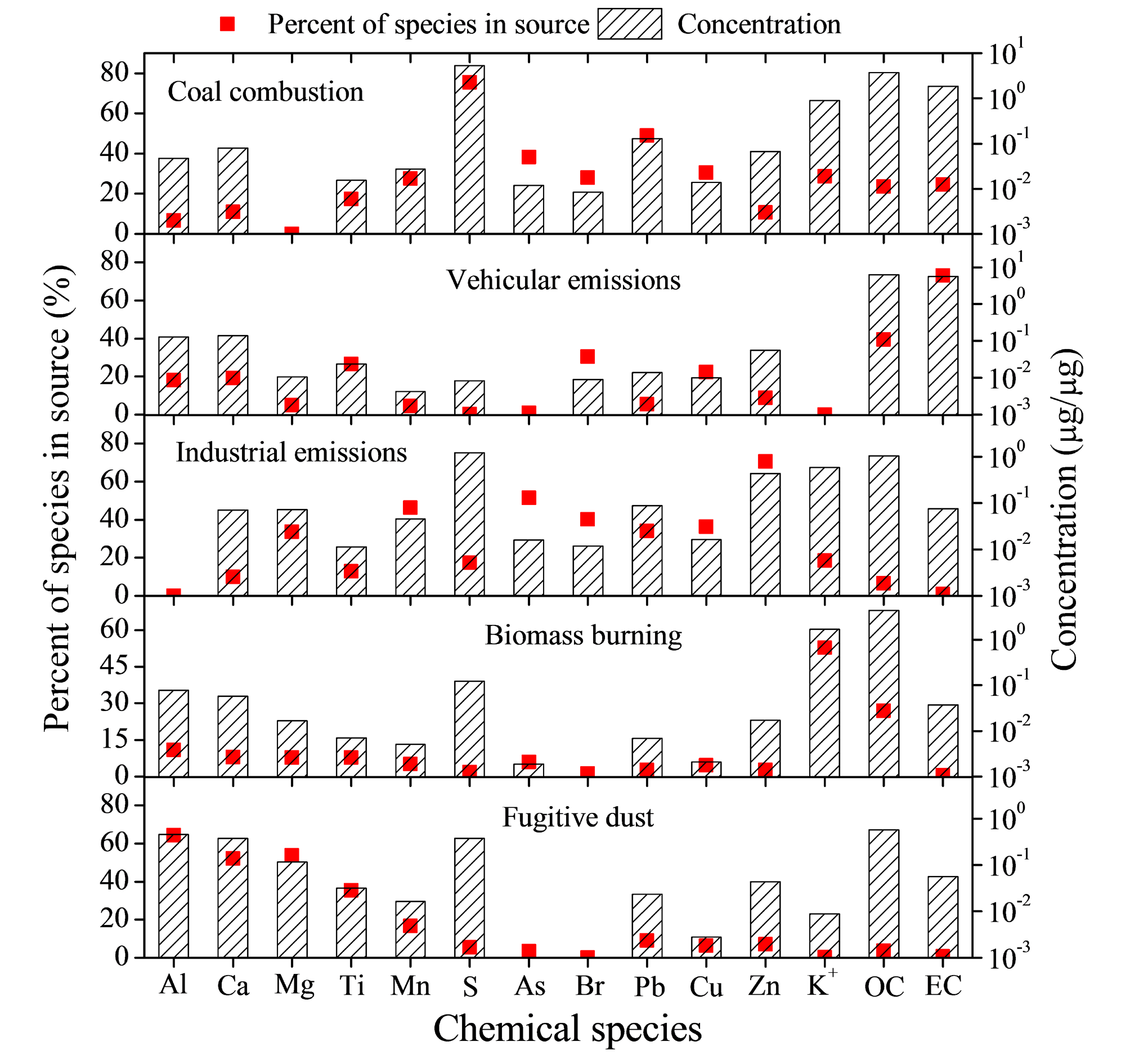

Supplement: Figure S4 — Source profiles for the five sources identified by the Positive Matrix Factorization (PMF) model during the intensive sampling period at Chengdu. Left Y-axis represents the percentage that each source contributes to each species. Right Y-axis represents the relative concentration that each source contributes to the species. (TIF) [file pone.0068894.s004.tif]
