# Supplementary material for: Long-Term Trends in Visibility and at Chengdu, China
Source: PLoS One. 2013 Jul 18;8(7):e68894. doi: 10.1371/journal.pone.0068894 (PMC3715545; doi:10.1371/journal.pone.0068894)
Supplement: Table S1 — Summary of the concentrations and signal-to-noise ratios (S/N) for the analytes used in PMF analysis. (DOCX) [file pone.0068894.s005.docx]

Table S1 Summary of the concentrations and signal-to-noise ratios (S/N) for the analytes used in PMF analysis.

| Analyte^a^ | Number | Mean | SD^b^ | S/N^c^ |
| --- | --- | --- | --- | --- |
| Al | 112 | 0.75 | 0.88 | 3.86 |
| Ca | 112 | 0.72 | 0.60 | 44.06 |
| Mg | 111 | 0.31 | 0.24 | 0.80 |
| Ti | 112 | 0.09 | 0.06 | 6.36 |
| Mn | 112 | 0.10 | 0.06 | 3.63 |
| S | 112 | 7.07 | 4.30 | 30.90 |
| As | 95 | 0.04 | 0.02 | 0.75 |
| Br | 105 | 0.04 | 0.04 | 1.07 |
| Pb | 112 | 0.27 | 0.15 | 7.00 |
| Cu | 112 | 0.05 | 0.03 | 2.42 |
| Zn | 112 | 0.63 | 0.41 | 38.76 |
| K^+^ | 112 | 3.20 | 1.96 | 43.92 |
| OC | 112 | 16.64 | 8.95 | 8.46 |
| EC | 112 | 7.86 | 5.40 | 7.59 |

^a^Units for analytes: μg m^-3^.

^b^S.D.: Standard deviation.

^c^
