# Supplementary material for: Long-Term Trends in Visibility and at Chengdu, China
Source: PLoS One. 2013 Jul 18;8(7):e68894. doi: 10.1371/journal.pone.0068894 (PMC3715545; doi:10.1371/journal.pone.0068894)
Supplement: Table S2 — Visual ranges (VR) for selected cities in China. (DOCX) [file pone.0068894.s006.docx]

Table S2 Visual ranges (VR) for selected cities in China.

| Location | Observation period | VR (km) | Reference |
| --- | --- | --- | --- |
| Chengdu | 1973-2010 | 8.5 | This study |
| Beijing | 1973-2007 | 10.7 | [28] |
| Guangzhou | 1973-2007 | 10.8 | [28] |
| Shanghai | 1973-2007 | 8.6 | [28] |
| Shenyang | 1973-2007 | 8.2 | [28] |
| Xi'an | 1995-2007 | 9.7 | [28] |
| Changzhou | 1981-2005 | 23.3 | [11] |
| Nanjing | 1981-2005 | 16.5 | [11] |
| Hangzhou | 1981-2005 | 15.1 | [11] |
| Tianjin | 1980-2008 | 13.6 | [29] |
| Hebei | 1980-2008 | 18 | [29] |
